# Metastatic prostate cancer with neuroendocrine differentiation and liver metastases: prognosis and response to platinum-based chemotherapy

**DOI:** 10.1007/s00345-026-06859-3

**Published:** 2026-10-28

**Authors:** Christian W. Schaaf, G. von Amsberg, J. J. Werner, C. Mogler, N. Borcherding, N. Pfarr, K. Steiger, F. Jungmann, L. Steinhelfer, T. Lorenzini, U. A. Schatz, J. Steinhaus, L. Renders, U. Heemann, J. Gschwend, M. Retz, M. Jahnen

**Affiliations:** 1 School of Medicine and Health, Department of Nephrology, Technical University of Munich, TUM University Hospital, Ismaninger Str. 22, 81675 Munich, Germany; 2 Department of Hematology and Oncology, Martini‑Klinik, Prostate Cancer Center, University Cancer Center Hamburg, University Medical Center Hamburg- Eppendorf, University Medical Center Hamburg-Eppendorf, Martinistr. 52, 20246 Hamburg, Germany; 3 School of Medicine and Health, Department of Urology, Technical University of Munich, TUM University Hospital, Munich, Germany; 4 School of Medicine and Health, Institute of Pathology, Technical University of Munich, Munich, Germany; 5 School of Medicine and Health, Department of Diagnostic and Interventional Radiology, Technical University of Munich, TUM University Hospital, Munich, Germany; 6 Department of Diagnostic and Interventional Radiology and Nuclear Medicine, University Medical Center Hamburg- Eppendorf, Hamburg, Germany; 7 School of Medicine and Health, Department of Nuclear Medicine, Technical University of Munich, TUM University Hospital, Munich, Germany; 8 German Cancer Consortium (DKTK), partnersite Munich, German Cancer Research Center (DKFZ), Heidelberg, Germany; 9 TUM School of Medicine and Health, Gynecological Tumor Genetics, Technical University of Munich, TUM University Hospital, Munich, Germany

**Keywords:** Neuroendocrine prostate cancer, Liver metastasis, Platinum-based chemotherapy

## Abstract

**Purpose:**

Neuroendocrine differentiation represents an aggressive prostate cancer subtype with poor prognosis. Platinum-based chemotherapy (PT) is standard of care, yet responses are often short-lived. This study aimed to define histopathological subgroups and identify predictors of survival in patients with liver-metastatic prostate cancer with neuroendocrine differentiation treated with PT.

**Methods:**

We retrospectively analyzed 950 patients with metastatic prostate cancer treated between 2006 and 2025, identifying 31 patients with neuroendocrine differentiation, liver metastases and PT. Tumors were classified into four subgroups based on the proportion of neuroendocrine features. Clinical characteristics, treatment response, tumor markers, and molecular alterations (*TP53*,* RB1*,* PTEN*,* BRCA1/2*) were evaluated. Overall survival (OS) was analyzed using Kaplan–Meier estimates and Cox regression.

**Results:**

Median OS following PT initiation was 8.5 months. Disease control was achieved in 81% of patients, but responses were transient. Patients with a high proportion of remaining adenocarcinoma features showed the longest median OS (15 months), while pure neuroendocrine subtypes had the poorest outcomes (6–10 months). Osteolytic bone metastases (HR 2.48; 95% CI 1.08–5.72), *TP53* loss-of-function variants (HR 2.43; 95% CI 1.14–5.16), and ≥ 2 concurrent alterations in *TP53/RB1/PTEN* (HR 2.13; 95% CI 1.03–4.41) were associated with inferior OS. Early reduction in liver metastasis size (HR 0.41; 95% CI 0.19–0.89) and decline in LDH (HR 0.50; 95% CI 0.23–1.09) after two treatment cycles predicted improved survival.

**Conclusions:**

Metastatic prostate cancer with neuroendocrine differentiation and liver metastases is characterized by extreme aggressiveness. There seems to be only limited durable benefit from PT especially in the presence of pure neuroendocrine morphology and tumor suppressor gene alterations. Therefore, these characteristics seem to be promising criteria to be investigated in larger prospective trials to further validate their clinical implications.

## Introduction

Metastatic prostate cancer is among the most common metastatic malignancies worldwide [1]. Modern treatment strategies have improved outcomes for most patients with acinar adenocarcinoma. A distinct subgroup of metastatic prostate cancer with neuroendocrine differentiation still faces limited therapeutic options and a markedly worse prognosis [2–4]. Neuroendocrine differentiation in metastatic prostate cancers may occur de novo or emerge during treatment. Especially the incidence of such therapy-induced neuroendocrine differentiation is increasing, driven by treatment-induced lineage plasticity and transdifferentiation of adenocarcinoma cells into neuroendocrine phenotypes under therapeutic pressure [4–7]. This transdifferentiation is often a dynamic and fluid process, resulting in tumors that frequently contain both adenocarcinoma and neuroendocrine components. Accordingly, the World Health Organization´s definition for prostate cancer with neuroendocrine differentiation comprises several histological entities, including pure small-cell and large-cell neuroendocrine carcinoma, adenocarcinoma with neuroendocrine differentiation, rare Paneth cell-like variants, well-differentiated carcinoid tumors, and mixed neuroendocrine carcinoma–acinar adenocarcinoma [4, 8]. Clinically, the presence of either pure or partial neuroendocrine differentiation is determinant of the disease course and associated with an increased incidence of visceral metastases, reduced sensitivity to standard prostate cancer therapies, and poor overall survival [2, 9, 10]. 

Platinum-based chemotherapy is the recommended treatment in these cases, particularly for therapy-induced neuroendocrine differentiation and small-cell variants. However, responses are typically short-lived, with median progression-free survival rarely exceeding several months and persistently poor overall survival [9, 11–13]. Still, the therapeutic efficacy seems to vary substantially across cases, but predictors of treatment response remain insufficiently defined. Addressing this gap, our retrospective study aimed to delineate clinically relevant histopathological and clinical subgroups of metastatic prostate cancer with neuroendocrine differentiation and identify predictors of response in such patients with liver metastases treated with platinum-based chemotherapy.

## Patients and methods

### Cohort

**Fig. 1:**
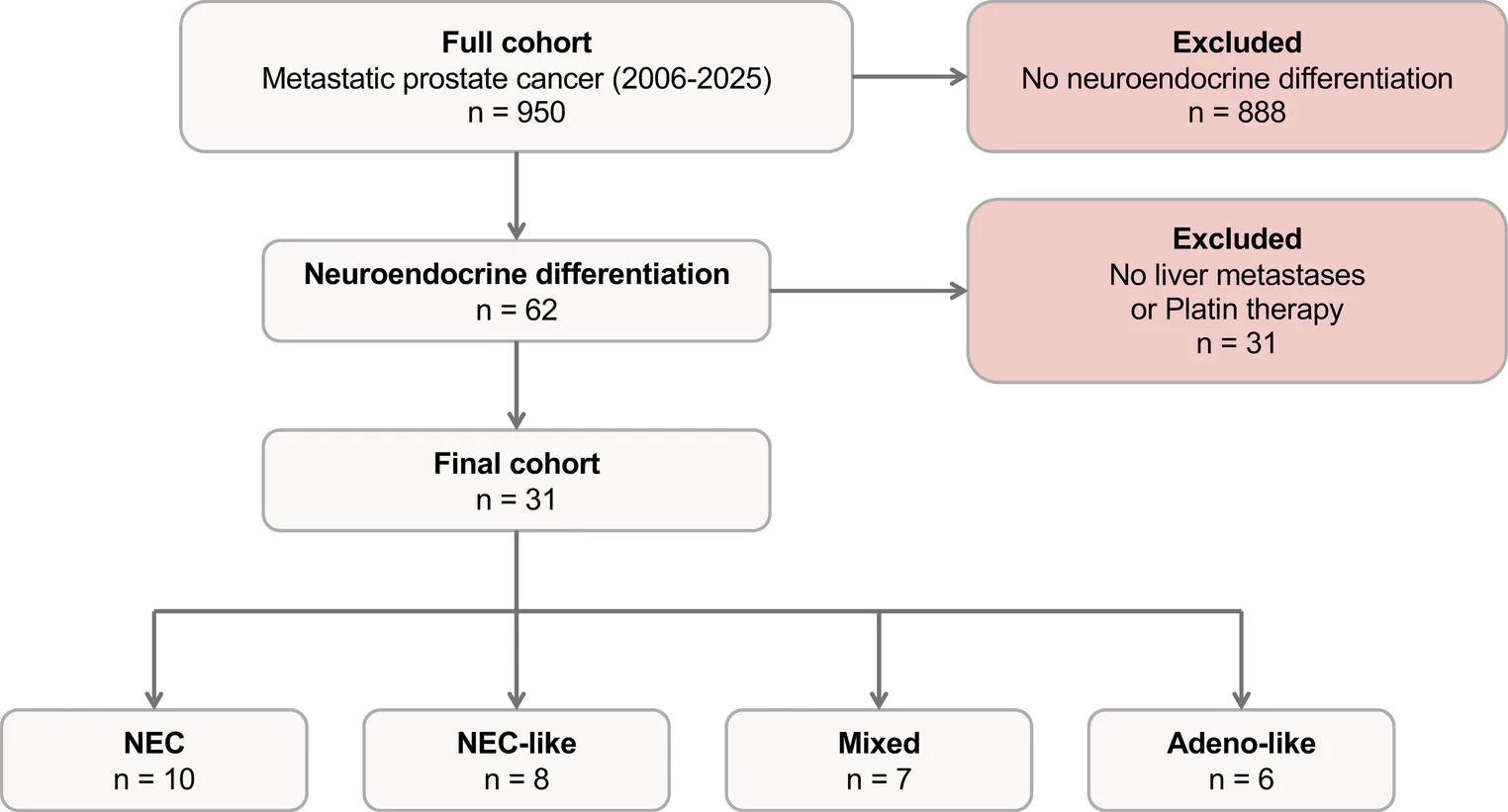
Flowchart of included patients with metastatic prostate cancer and neuroendocrine differentiation

We retrospectively analyzed a cohort of 950 patients with metastatic prostate cancer, who were treated between 2006 and 2025 at the Department of Urology, TUM University Hospital, Munich, Germany. All cases were evaluated for histologically confirmed pure or partial neuroendocrine differentiation (Fig. 1). The study was conducted in accordance with the Declaration of Helsinki and the Guidelines for Good Clinical Practice, and was approved by the local institutional review board (2024-143-S-SB).

Neuroendocrine differentiation was defined according to WHO criteria and established published definitions featuring either pure small-cell or large-cell neuroendocrine carcinoma (NEC) or conventional NEC [5, 8]. For the present analysis, the cohort was classified into five predefined histological subgroups, representing the spectrum of increasing neuroendocrine expression and decreasing adenocarcinoma features:

Group 1 (Pure neuroendocrine expresser): Exclusive neuroendocrine features without an adenocarcinoma component and with predominantly solid growth and high proliferation, including small-cell and large-cell NEC. Group 2 (Double expresser, NEC-like): Predominantly neuroendocrine features (> 80%) with solid growth and co-expression of neuroendocrine markers (synaptophysin, chromogranin A [CGA]) and adenocarcinoma-typical markers (PSA, NKX3.1).

Group 3 (Double expresser, mixed): Mixed adeno-neuroendocrine carcinomas exhibiting both solid neuroendocrine and adenocarcinoma markers, either intermixed within the tumor or present as adjacent, distinct regions, each showing its respective typical immunophenotype.

Group 4 (Double expresser, adenocarcinoma-like): Adenocarcinoma-like tumors with predominantly glandular morphology (> 80%) and co-expression of neuroendocrine (Synaptophysin/CGA) and adenocarcinoma-associated markers (PSA/NKX3.1).

Group 5 (Pure adenocarcinoma expresser): Conventional adenocarcinomas without histological or immunohistochemical evidence of neuroendocrine differentiation.

### Clinical characteristics

The following clinical variables were extracted from medical records: age at diagnosis of neuroendocrine differentiation; onset of neuroendocrine differentiation (de novo vs. therapy-induced); baseline serum tumor markers at initiation of platinum-based chemotherapy, including prostate-specific antigen (PSA), lactate dehydrogenase (LDH), CGA, and neuron-specific enolase (NSE); history of radical prostatectomy; number and type of systemic therapies administered prior to platinum-based chemotherapy (androgen-deprivation therapy, antiandrogens, taxane-based chemotherapy, radioligand therapy); presence of lymph node metastases; presence and pattern of bone metastases (osteolytic vs. osteoblastic); presence of liver metastases; and number of cycles of platinum-based chemotherapy.

### Biomarkers

Biomarker profiling was performed through retrospective review of molecular pathology reports, supplemented by renewed microscopic evaluation by an experienced pathologist. The following parameters were assessed for all patients: Immunohistochemical expression of PSA, NKX3.1, synaptophysin, chromogranin A, and MIB1 (Ki-67). Molecular genetic alterations in *TP53*,* RB1*,* PTEN*, and *BRCA1/2*. Genomic data were generated using TruSight Oncology 500 and Oncomine Comprehensive next-generation sequencing (NGS) gene panels. Variants with a variant allele frequency ≥ 5% were included in the analysis, provided that the predefined assay-specific coverage thresholds were met: >150× for hybrid-capture panel sequencing. Variants outside coding exons and the ± 10-base pair splice regions, synonymous variants, and variants with a population allele frequency > 1% in Single Nucleotide Polymorphisms or the Genome Aggregation Database were excluded.

### Tumor response

Response to platinum-based chemotherapy was evaluated according to RECIST 1.1. criteria. For each patient, the best overall radiological response to platinum-based treatment at any staging timepoint was recorded. Additionally, changes in the extent of liver metastases after two treatment cycles of platinum-based chemotherapy were specifically assessed. Laboratory response was evaluated by analyzing changes in PSA, LDH, CGA, and NSE levels after two cycles of platinum-based chemotherapy.

### Statistical analysis

Descriptive statistics were calculated for all study variables. Differences between histological subgroups were assessed using Chi-square tests for categorical variables and Wilcoxon rank-sum tests for continuous variables. Univariate Cox regression models were used to identify clinical and molecular predictors of overall survival after initiation of platinum-based therapy. Kaplan–Meier estimates and log-rank tests were applied to compare overall survival in evaluable patients stratified by histological subgroups and clinically relevant covariates identified in regression analyses. All statistical tests were two-sided with a significance level of α = 0.05. Analyses were performed using SPSS 29.0 (IBM Corporation, Armonk, NY, USA) and R.

## Results

### Clinical, histopathological, and molecular characteristics

Among 950 assessed mCRPC patients, 62 were identified with histologically confirmed neuroendocrine differentiation. Of these, 31 patients had liver metastases and received platinum-based chemotherapy (30/31 cisplatin/etoposide; 2/31 carboplatin/etoposide) with a median of four treatment cycles. For comparison, six patients with pure adenocarcinoma, liver metastases, and no evidence of neuroendocrine differentiation who received platinum-based chemotherapy (6/6 cisplatin/etoposide) were included as a control group.

Of the 31 patients with neuroendocrine differentiation, 4 (13%) presented with neuroendocrine features at primary diagnosis, with two of these cases being pure small-cell NEC. All other 27 patients (87%) developed neuroendocrine features during the disease course. The median time from initial diagnosis to the biopsy-proven detection of either pure (8/27) or partial (19/27) neuroendocrine differentiation among these patients was 25 months (interquartile range: 12–52). Additional clinical characteristics are summarized in Table 1.

**Table 1:** Clinical characteristics of all patients and according to histological subgroups

Group Number	1 (*n* = 10)	2 (*n* = 8)	3 (*n* = 7)	4 (*n* = 6)	5 (*n* = 6)	All (*n* = 37)
Group description	NEC	NEC-like	Mixed	Adeno-like	Adeno	
Age at first diag. of neuroendocrine diff., mean (SD) *years*	64.6 (6.0)	66.8 (7.6)	69.6 (11)	67.2 (8.6)	65.5 (7.4)	66.5 (7.7)
PSA at first diag. of neuroendocrine diff., med (IQR) *ng/ml*	0.4 (0.04–16)	5.5 (0.5–29)	58 (8.1–110)	19 (11–95)	27 (2.5–221)	9.0 (0.4–107)
NSE at first diag. of neuroendocrine diff., med (IQR) *ng/ml*	61 (51–66)	19 (17–38)	39 (34–550)	23 (14–32)	24 (18–31)	33 (19–62)
CGA at first diag. of neuroendocrine diff., med (IQR) *ng/ml*	155 (92–203)	128 (83–195)	312 (179–725)	424 (151–571)	230 (120–263)	180 (90–420)
LDH at first diag. of neuroendocrine diff., (IQR) *U/l*	437 (259–571)	267 (251–288)	299 (284–983)	217 (202–315)	563 (385–894)	299 (254–571)
Prostatectomy, n (%)	5 (50)	4 (50)	4 (57)	5 (71)	6 (100)	24 (65)
Lymphogenic metastasis, n (%)	10 (100)	7 (88)	6 (86)	5 (3)	6 (100)	34 (92)
Bone metastasis, n (%)	10(100)	8 (100)	6 (86)	4 (67)	5 (83)	33 (89)
Osteolytic metastasis, n (%)	5 (50)	2 (25)	1 (14)	1 (17)	1 (17)	10 (27)
Visceral metastasis (other than liver)	6 (60)	4 (50)	3 (43)	2 (33)	1 (17)	16 (43)
Primary liver metastasis, n (%)	7 (70)	6 (75)	5 (71)	2 (33)	0 (0)	20 (65)
Neuroendocrine histology, n (%)						
De novo	2 (20)	1 (13)	1 (14)	0 (0)	–	4 (13)
Therapy induced	8 (80)	7 (87)	6 (86)	6 (100)	–	27 (87)
Platinum-based therapy						
Number of cycles, med (IQR)	4.5 (4–6)	5 (4–6.5)	6 (2.5–6)	5 (4–6)	3 (2–5.5)	4 (3–6)
Best radiological response, n (%)						
PD	2 (20)	1 (13)	2 (29)	0 (0)	2(34)	7 (19)
SD	3 (30)	3 (38)	1 (14)	4 (67)	3 (50)	14 (38)
PR	5 (50)	4 (50)	4 (57)	2 (33)	1 (17)	16 (43)
CR	0 (0)	0 (0)	0 (0)	0 (0)	0 (0)	0 (0)
Therapy lines before platinum-based therapy						
Number of therapy lines, med (IQR)	2 (2–3)	3 (2–4.25)	4 (2.5–4.5)	4.5 (3.25–5)	4 (4–4)	3 (2–4)
Type of therapies, n (%)						
Androgen receptor pathway inhibitor (ARPI)	8 (80)	8 (100)	6 (86)	6 (100)	6 (100)	34 (92)
Second ARPI	6 (60)	7 (88)	6 (86)	6 (100)	6 (100)	31 (84)
Lutetium-177-PSMA	1 (10)	3 (38)	4 (67)	3 (50)	2 (33)	13 (36)
Docetaxel	5 (50)	4 (50)	3 (43)	3 (50)	6 (100)	21 (57)
Cabazitaxel	0 (0)	2 (25)	2 (29)	3 (50)	6 (100)	13 (36)

Group 1 (Single neuroendocrine expresser): neuroendocrine carcinoma without an adenocarcinoma component; Group 2 (Double expresser, NEC-like): neuroendocrine carcinoma-like with predominantly neuroendocrine markers and some adenocarcinoma-typical markers; Group 3 (Double expresser, mixed): Mixed adeno-neuroendocrine carcinomas; Group 4 (Double expresser, adenocarcinoma-like): Adenocarcinoma-like with co-expression of some neuroendocrine markers (PSA/NKX3.1); Group 5 (Single adenocarcinoma expresser): Conventional adenocarcinomas without neuroendocrine differentiation

*Diag.* diagnosis, *diff.* differentiation, *SD* standard deviation, *IQR* interquartile range, *Diag.* diagnosis, *med* median, *PSA* prostate specific antigen, *LDH* lactate dehydrogenase, *CGA* chromogranin A, *NSE* neuron-specific enolase, *PD* progressive disease, *SD* stable disease, *PR* partial response, *CR* complete response

The 31 patients were further subclassified based on histological and molecular features: group 1: pure neuroendocrine expresser, (NEC, *n* = 10, 32%); group 2: double expresser (NEC-like, *n* = 8, 26%); group 3: double expresser, (mixed, *n* = 7, 23%) and group 4: double expresser, (adenocarcinoma-like, *n* = 6, 19%).

In contrast to the mixed and adenocarcinoma-like groups, patients with NEC and NEC-like tumors had a high incidence of primary liver and other visceral metastases at the time of initial diagnosis, as well as osteolytic bone metastases at the time of diagnosis of neuroendocrine differentiation (Table 1).

**Fig. 2:**
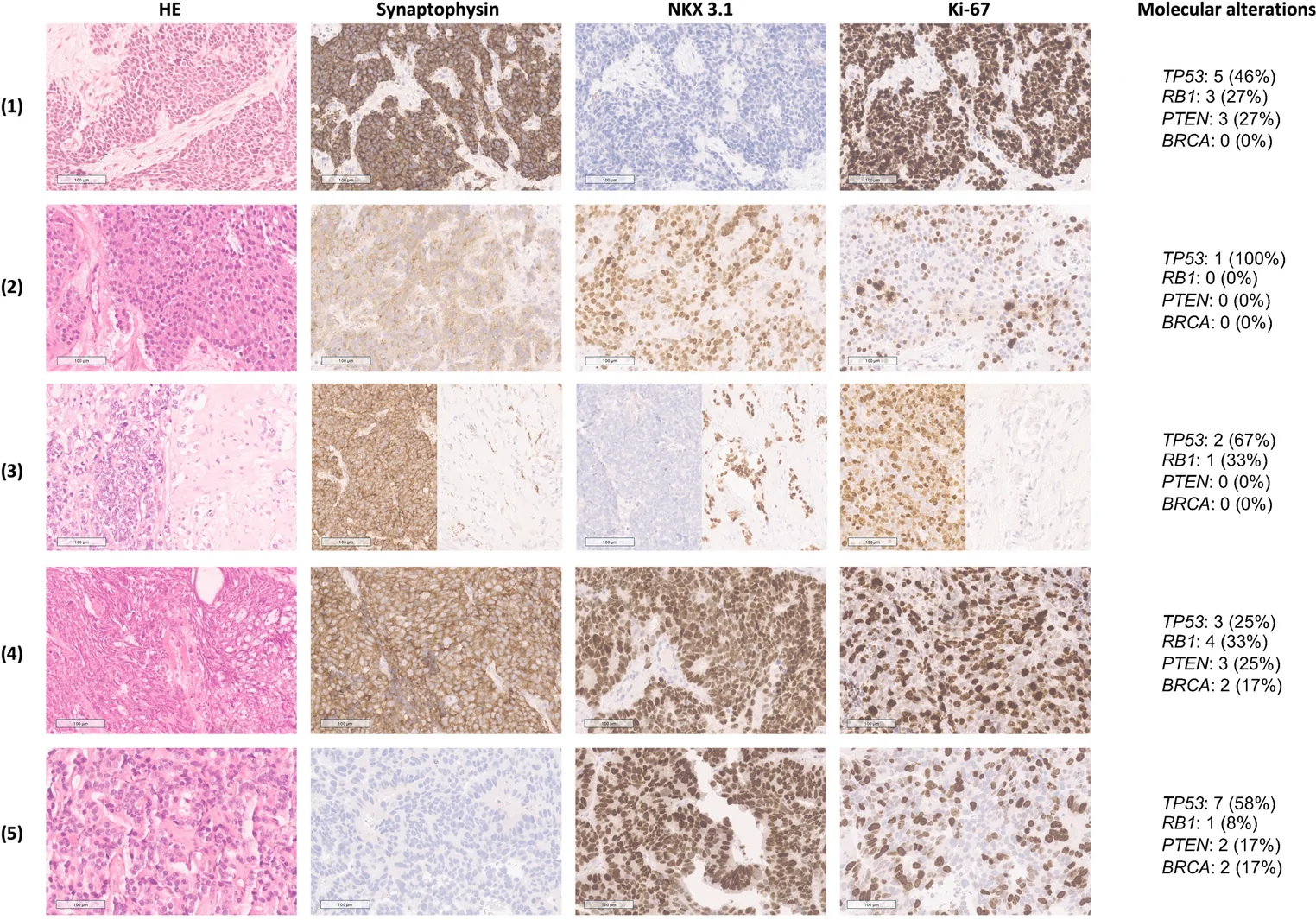
Representative histology (hematoxylin and eosin staining) and immunohistochemistry for synaptophysin, NKX3.1, and Ki-67/MIB-1 across molecularly defined groups with molecular alterations within each group indicated on the right side. (1) Pure neuroendocrine expresser; (2) Double expresser (NEC-like); (3) Double expresser, mixed; (4) Double expresser, adenocarcinoma-like; (5) Pure adenocarcinoma expresser. NKX3.1 = NK3 homeobox 1; Ki-67 = proliferation marker protein Ki-67; TP53 = tumor protein p53; RB1 = RB transcriptional corepressor 1; PTEN = phosphatase and tensin homolog; BRCA = breast cancer gene (BRCA1/2); (1) = NEC; (2) = NEC-like, (3) = Mixed; (4) = Adeno-like; (5) = Adeno

Molecular data was available in 28 of 31 patients (90%) and was based primarily on tissue extracted from liver metastases (86%) or alternatively the primary tumor (14%). In all cases molecular pathological analysis was carried out prior to the start of platinum-based chemotherapy. In patients with NEC- (group 1) and NEC-like (group 2) subtypes, loss-of-function variants in *TP53* and pathogenic alterations in *RB1* and *PTEN* were observed in 55%, 44% and 50%, respectively. This was associated with marked proliferative activity (Ki-67 > 80%) and strong expression of neuroendocrine markers such as synaptophysin and chromogranin A (CGA). In contrast, patients within the mixed (group 3) and adenocarcinoma-like (group 4) subtypes exhibited lower frequencies of *RB1* (23%) and *PTEN* (30%) alterations, while *TP53* (69%) alterations were also common. The control group (group 5) demonstrated alterations in these tumor suppressor genes in less patients (*TP53* 16%, *RB1* 32%, *PTEN* 16%), with generally low Ki-67 indices and absent of neuroendocrine marker expression. Representative examples of marker expression patterns in the different groups are presented in Fig. 2.

### Response to platinum-based chemotherapy

**Fig. 3:**
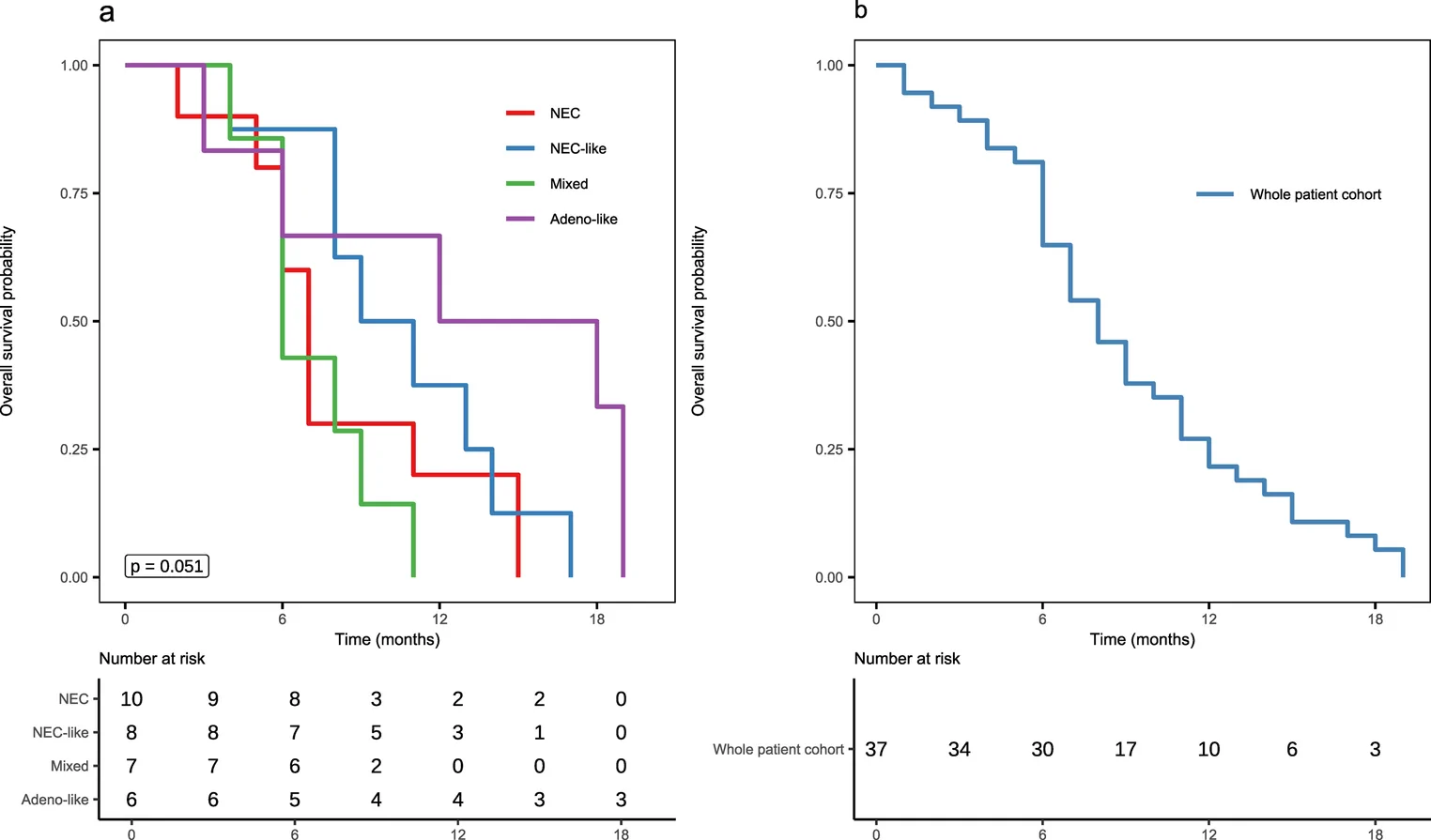
Overall survival from the start of platin-based chemotherapy, stratified by histological subgroups (**a**) and the whole patient group (**b**)

As the best measured response under platinum-based chemotherapy, in patients with neuroendocrine differentiation the disease control rate (DCR) defined as stable disease or a partial response was 81%, while 19% experienced immediate disease progression. In the adenocarcinoma group, the DCR was 67%. No complete response (CR) was achieved in any of the groups. After the last cycle of treatment, additional 54% of patients with neuroendocrine differentiation and 50% of adenocarcinoma patients showed disease progression. The median overall survival (OS) following initiation of platinum-based chemotherapy was 8.5 months (95% CI: 6.5–12) among patients with neuroendocrine differentiation and 8 months (95% CI: 1–12) in the adenocarcinoma group. Among the four histological subgroups with neuroendocrine features, the NEC, NEC-like, and mixed subgroups showed short median overall survival after initiation of platinum-based chemotherapy (7, 10, and 6 months, respectively), whereas the adenocarcinoma-like subgroup demonstrated the longest median overall survival (15 months) (Fig. 3).

**Fig. 4:**
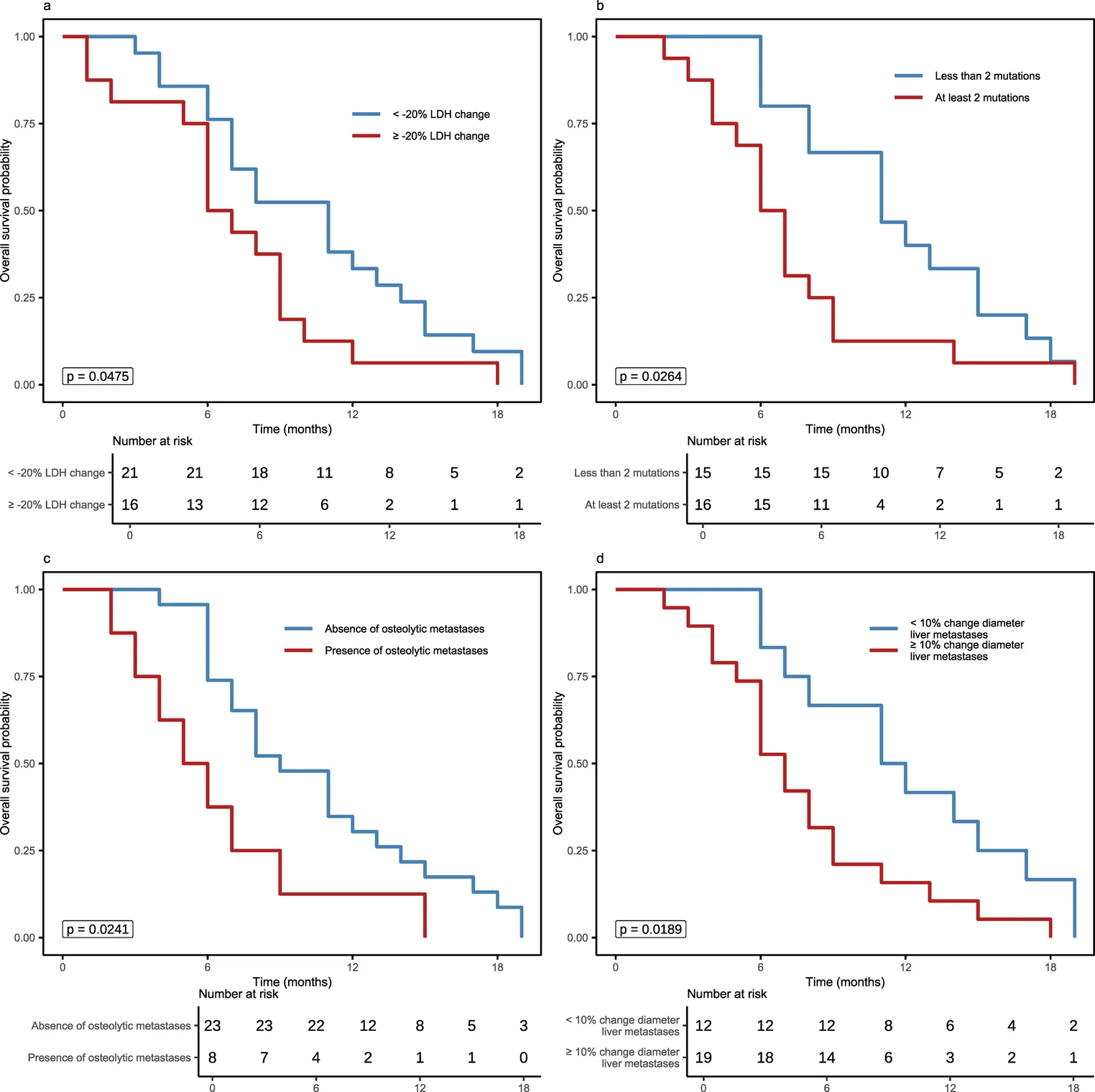
Overall survival from the start of platinum-based chemotherapy based on LDH change after two cycles (**a**), combined mutation status in *TP53*,* RB1*, and *PTEN* (≥ 2 mutations vs. <2 mutations) (**b**), presence of osteolytic metastasis (**c**), and radiographic change in the sum diameter of liver metastases after two cycles (**d**)

Focusing on clinical baseline characteristics, univariate Cox regression analysis identified presence of osteolytic metastases (HR 2.48; 95% CI 1.08–5.72; *p* = 0.03) significantly associated with poorer outcomes (Table 2; Fig. 4). Moreover, a reduction in liver metastasis size by at least 10% (HR 0.41; 95% CI 0.19–0.89; *p* = 0.03) and a ≥ 20% decline in LDH (HR 0.50; 95% CI 0.23–1.09; *p* = 0.03) after two cycles of platinum-based chemotherapy were an independent predictor of improved overall survival.

**Table 2:** Clinical, molecular, pathological and biochemical characteristics associated with worse overall survival under cisplatin-based chemotherapy in univariate Cox regression analysis

Characteristics	HR (95% CI)	*p*-value
Treatment-emergent neuroendocrine differentiation	0.57 (0.27–1.21)	0.1
Predominantly NEC histology (Group 1 + 2)	3.48 (0.98–12.35)	**0.04**
Osteolytic metastasis	2.48 (1.08–5.72)	**0.03**
*TP53* LOF Mutation	2.43 (1.14–5.16)	**0.02**
≥ 2 pathogenic variantes in *PTEN/TP53/RB1*	2.13 (1.03–4.41)	**0.04**
PSA (pre-cisplatin-based chemotherapy) ≥ 20 ng/ml	0.90 (0.44–1.87)	0.8
LDH (pre-cisplatin-based chemotherapy) ≥ 488 ng/ml	1.78 (0.77–4.11)	0.2
CGA (pre-cisplatin-based chemotherapy) ≥ 300 ng/ml	0.78 (0.37–1.64)	0.5
NSE (pre-cisplatin-based chemotherapy) ≥ 40 ng/ml	1.29 (0.63–2.64)	0.5
LDH change (after two cycles) ≥ 20%	0.50 (0.23–1.09)	**0.03**
Diameter of liver metastasis change (after two cycles) > 0%	0.38 (0.16–0.93)	**0.03**

* LOF* Loss of function, *PSA* prostate specific antigen, *LDH* lactate dehydrogenase, *CGA* chromogranin A, *NSE* neuron specific enolase

HR and 95% CI were derived from univariate Cox regression; p-values were calculated using the log-rank test. Bold values indicate statistical significance (p<0,05)

Analysis of genomic alterations further revealed that *TP53* loss-of-function variants (HR 2.43; 95% CI 1.14–5.16; *p* = 0.02) and ≥ 2 concurrent pathogenic molecular events (*TP53*,* RB1*,* PTEN*) (HR 2.13; 95% CI 1.03–4.41; *p* = 0.04) correlated with worse overall survival.

## Discussion

De novo and therapy-induced neuroendocrine differentiation represent the most aggressive end of the metastatic prostate cancer spectrum. In particular, the presence of liver metastases is strongly associated with poor overall survival and very limited treatment options [2, 4, 7, 9]. Data from our analysis further underline the profound aggressiveness of this disease entity. Median overall survival following initiation of platinum-based chemotherapy for hepatically metastasized prostate cancer with neuroendocrine differentiation in our cohort was only 8.5 months.

These results are comparable to survival outcomes reported in other cohort studies and larger registers of metastatic prostate cancer with neuroendocrine differentiation, which range between 9 and 16.8 months [3, 7, 10, 12, 14]. However, most published studies report survival from initial prostate cancer diagnosis or from first diagnosis of neuroendocrine differentiation, thereby limiting direct comparability [4, 10, 12].

In patients with metastatic prostate cancer and neuroendocrine differentiation, cisplatin in combination with etoposide remains the standard treatment of choice. In our cohort, 80% of patients achieved clinical disease control, including stable disease in 38% and partial response in 42% according to RECIST criteria, at least once during platinum-based treatment. However, after completion of four to six chemotherapy cycles, nearly 90% of patients experienced disease progression. This transient benefit aligns with previously published data. Papandreou et al. reported an objective response rate of 61% in a phase II trial of doxorubicin, etoposide, and cisplatin in small-cell prostate cancer, but with a median progression-free survival of only 5.8 months [13]. Similarly, in a prospective phase II study of carboplatin and etoposide in anaplastic mCRPC, Flechon et al. observed a clinical control rate of only 31% with a median duration of 4 months [11]. Moreover, in a retrospective analysis by Apostolides et al., only 48% of patients achieved disease control, with a mean duration of 6 months [9].

Given the limited efficacy of current therapies and the generally poor prognosis, we systematically evaluated clinical, histopathological, and molecular factors to identify subgroups that may derive differential benefit from aggressive platinum-based treatment. Among clinical baseline characteristics, only the presence of osteolytic bone metastases was significantly associated with inferior overall survival. This finding is consistent with previous reports identifying osteolytic lesions as a hallmark of highly aggressive prostate cancer wit neuroendocrine differentiation [2, 15].

Baseline biochemical markers were not associated with overall survival following initiation of platinum-based chemotherapy. This contrasts with prior studies reporting worse outcomes in patients with elevated baseline LDH, NSE or CGA levels [5, 12, 16, 17]. However, those studies measured survival from diagnosis of neuroendocrine differentiation and did not uniformly include patients with liver metastases. By restricting our analysis to patients with liver metastases and measuring survival from the start of platinum therapy, we aimed to reduce heterogeneity and confounding related to disease burden at diagnosis. Taken together, our data suggest that the prognostic relevance of static neuroendocrine biomarker measurements may diminish as the disease evolves. In contrast, dynamic treatment-associated biomarkers showed prognostic relevance. A marked decline in LDH during therapy (median OS: 12 vs. 6 months), and a pronounced radiologic response (size decrease of ≥ 10%) of liver metastases (median OS: 11 vs. 6 months) after two cycles of platinum chemotherapy were associated with improved overall survival. These findings suggest that close biochemical and radiologic monitoring could potentially serve as surrogates to enable early treatment adaptation, minimize unnecessary toxicity, and facilitate timely integration of supportive care.

Patients were further stratified into four histological subgroups based on dominant growth patterns. Patients with a high proportion of remaining adenocarcinoma features demonstrated the most favorable outcomes, whereas those with pure neuroendocrine histology experienced the poorest treatment response and survival. These subtype-dependent differences are consistent with previous reports describing particularly poor outcomes in pure neuroendocrine and small-cell NECs [15, 18, 19]. Nevertheless, our data also indicate that liver metastases, which often represent the lesions most relevant to prognosis in metastatic prostate cancer, may be driven by tumor cells retaining adenocarcinoma features. To contextualize this finding within our cohort, we included patients with pure adenocarcinoma who similarly presented with liver metastases and received platinum-etoposid-based chemotherapy. Notably, these patients experienced similarly poor overall survival following treatment initiation. These findings suggest that liver metastases may represent a particularly aggressive manifestation of metastatic prostate cancer, irrespective of histological subtype. Importantly, recent data indicate that early combination therapy with platinum and a taxane rather than platinum-etoposid-based chemotherapy may achieve more substantial responses in aggressive prostate adenocarcinoma [20]. Collectively, these observations support consideration of a biopsy in patients with newly diagnosed or progressing liver metastases to characterize the predominant histological and molecular phenotype and help inform the selection of a more neuroendocrine- or adenocarcinoma-directed treatment strategy. This distinction may be particularly relevant when selecting an appropriate chemotherapy regimen and when considering the discontinuation of androgen-deprivation therapy in patients with histologically pure neuroendocrine differentiation without evidence of of residual androgen receptor–driven disease.

Molecular profiling further revealed marked differences across histological subgroups and strong correlations with clinical outcomes. Groups 1 and 2 (small-cell and large-cell NEC, NEC-like) exhibited the highest frequency of alterations in *TP53*, *RB1*, and *PTEN*, as well as high Ki-67 indices, while these alterations were significantly less frequent in Groups 3 and 4 (mixed and adenocarcinoma-like). Patients with pure metastatic adenocarcinoma (Group 5) showed the lowest prevalence of these genomic events. These findings align with experimental evidence demonstrating that combined loss of *RB1*, *TP53*, and *PTEN* drives lineage plasticity and aggressive neuroendocrine differentiation [3, 18, 21, 22]. Importantly, the presence of *TP53* loss-of-function variants, or the accumulation of two or more alterations among *TP53*, *RB1*, and *PTEN*, was associated with markedly inferior survival, with only 10% of patients alive beyond 12 months after initiation of platinum-based chemotherapy. This observation is consistent with prior analyses linking *TP53* variants to earlier development of liver metastases and shorter survival in metastatic prostate cancer with neuroendocrine differentiation [9, 21]. Moreover, *TP53* loss-of-function variants have been associated with primary platinum resistance in other malignancies, such as ovarian cancer [23].

Several limitations should be acknowledged. The retrospective study design and small sample size limited statistical power, as reflected by the wide confidence intervals for some estimates, particularly within the smaller molecular and histological subgroups. The limited number of patients and events also restricted the statistical assessment to univariable analyses, precluding adjustment for potential confounders such as prior treatments, performance status, and the extent of metastatic disease at other sites. By focusing exclusively on patients with liver metastases who received platinum-based chemotherapy, we sought to reduce clinical heterogeneity and establish a common therapeutic landmark. However, this selection further limits the generalizability of our findings to patients with neuroendocrine-differentiated prostate cancer without liver metastases or those who do not receive platinum-based chemotherapy. Overall, these results should be therefore considered hypothesis-generating and require validation in larger, preferably prospective studies. In addition, transitions between histological phenotypes are often fluid, as neuroendocrine and adenocarcinoma marker expression may vary across tumor regions and over time [19, 21]. Moreover, in 14% of patients, the assessment of genetic alterations was based on primary tumor tissue rather than liver metastasis tissue, further limiting its ability to accurately reflect the molecular profile at the initiation of platinum-based chemotherapy. Consequently, biopsy timing and sampling site should always be carefully considered when interpreting histological and molecular data. Finally, the absence of a control group precludes definitive conclusions regarding the true treatment effect of platinum-based chemotherapy, emphasizing the urgent need for prospective randomized trials investigating therapeutic strategies in metastatic prostate cancer with neuroendocrine differentiation.

## Conclusion

In summary, this study highlights the extreme aggressiveness and limited therapeutic options for patients with metastatic prostate cancer with neuroendocrine differentiation, particularly in the presence of liver metastases. These findings suggest that clinical management should carefully balance the modest and often transient benefits of potentially toxic platinum-based chemotherapy against the need for early supportive and palliative care. Robust biomarkers are required to better inform these complex clinical decisions. In this context, histological and molecular features, particularly the degree of neuroendocrine differentiation and alterations in *TP53*, *RB1*, and *PTEN*, as well as early biochemical and radiological responses represent promising candidates for prospective evaluation. In any case, biopsy of newly developed or progressing liver metastases should be considered in patients with mCRPC to enable accurate histopathological and molecular characterization.

## Data Availability

Data are available for bona fide researchers who request it from the authors.
